# The IFPA youth group, the Adolescent Confidential Telephone Service and Sexual Health Activism in Ireland, *c*. 1984–90

**DOI:** 10.1093/hwj/dbae018

**Published:** 2024-08-29

**Authors:** Laura Kelly

**Affiliations:** University of Strathclyde

**Keywords:** Ireland, sexual health, youth, activism, sexuality, counselling

## Abstract

In October 1984 the Irish Family Planning Association (IFPA) established a youth group of volunteers aged 16-20. One of the group’s main initiatives was a sexual health phoneline for young people called the Adolescent Confidential Telephone Service (ACTS). Using oral history interviews and archival sources such as the ACTS logbook, this article explores the motivations of the young activists involved in the ACTS and what the operation of and responses to it reveal about the wider social climate in relation to sexual health. Finally, it examines the emotional labour involved in sexual health activism, showing how this was often gendered.

## ***

**Figure 1.**
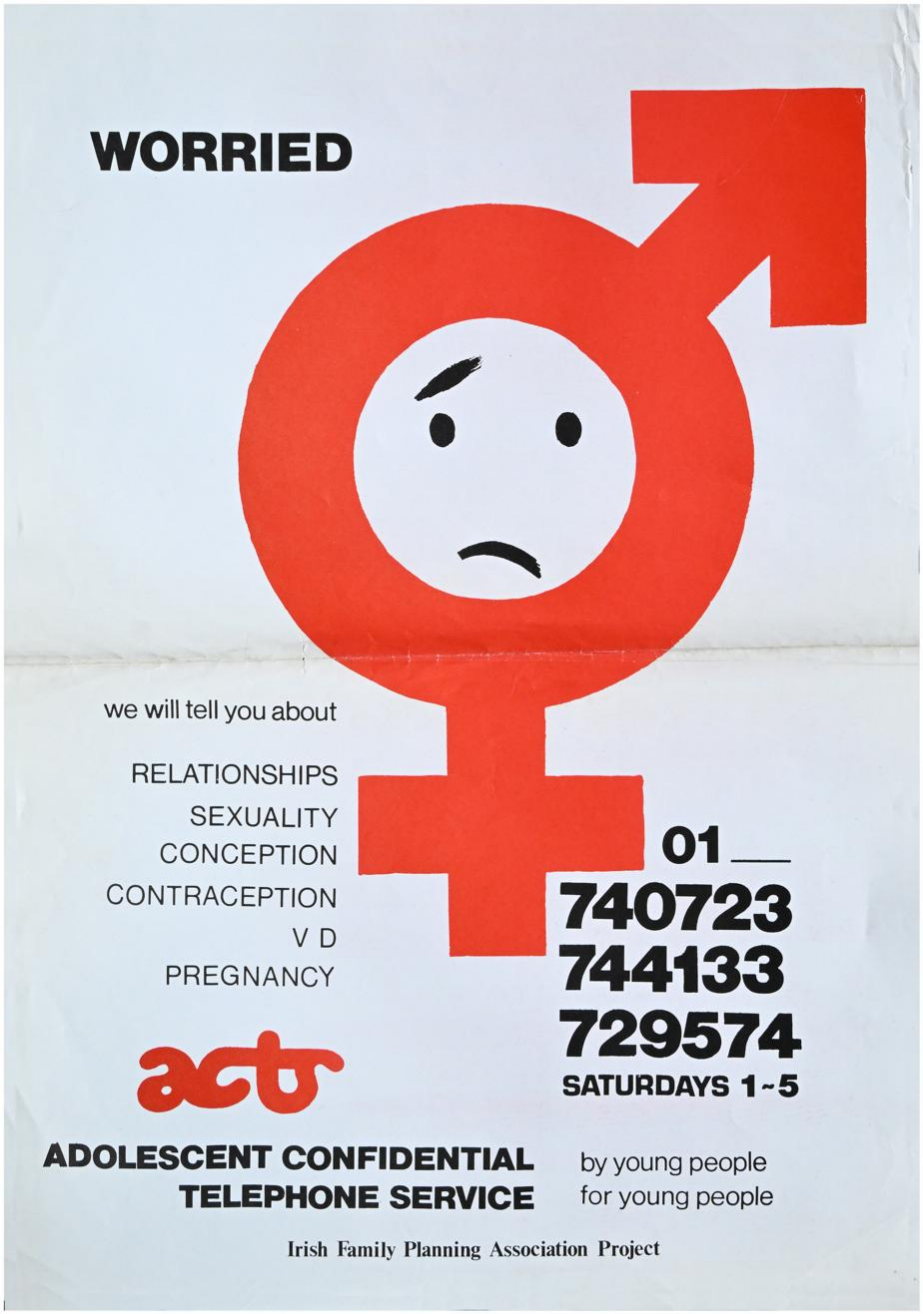
ACTS poster, 1985. Irish Family Planning Association archive, RCPI Heritage Centre, Dublin. With thanks to Harriet Wheelock.

On a Saturday afternoon in early November 1984, a group of young volunteers between the ages of sixteen and twenty began an afternoon of work on a unique new telephone line aimed at adolescents. The calls received in that first session were few and included numerous hang-ups, but after a few weeks of operation requests for information about sex and sexuality, access to contraception, relationship problems, unplanned pregnancy and cross-dressing, amongst other issues, began to trickle in.^1^ This telephone line, called the Adolescent Confidential Telephone Service (ACTS), was established by the Irish Family Planning Association (IFPA) youth group in Dublin in October 1984 (Figure 1). It operated on Saturday afternoons between 1 and 5 p.m. as a pilot project until December 1984, before being formally launched in January 1985 to mark International Youth Year, and it ran until July 1990. The youth group had been established by Christine Donaghy, then information and education officer, and Dr. Mary Short, an IFPA doctor.^2^ The group’s initiatives also included a Young People’s Family Planning Centre (set up in November 1986), problem pages in two magazines for young people, the production of educational leaflets and visits to local secondary schools. Their activism culminated in the ‘Virgin Condom Case’ (1988–91) where youth volunteers set up a counter in the Virgin Megastore in Dublin and sold condoms illegally in order to challenge the law around the sale of condoms.^3^

The social and legislative landscape that shaped attitudes towards reproductive rights and sexuality in Ireland was intimately entangled with Catholic teachings and clouded by feelings of shame and stigma.^4^ The 1980s and early 90s have tended to be characterized as a time of repression in Ireland, particularly in terms of reproductive rights.^5^ The period saw a significant backlash against measures in support of sexual liberation, including divisive referendums over divorce (1986, 1995) and abortion (1983, 1992).^6^ Homosexual acts were not decriminalized until 1993. Reflecting on the broader context and the important gap that the ACTS filled in providing sexual health information to young people who may have been too embarrassed or too young to seek help from other sources, former volunteer John Callaghan explained:

Of course, at the time you couldn’t, your local library didn’t stock this information. The chemist could have been a conservative, religious person who didn’t want to talk about these things with you or didn’t want to be dispensing contraceptive pills or whatever, where did you go for the information? Where did you go to look for it? It wasn’t available, literally it wasn’t available. It was very difficult for people to access it unless they would come, often people outside of Dublin would be making trips to Dublin or to Cork or to Galway or whatever, to get into a family planning centre … Then they had to deal with the shame and embarrassment and inhibition that traditional church teaching and their communities have put on them. Not having the language, not knowing what questions to ask. Being terrified that by accident their priest will be there, or their neighbours will be on the street outside and everybody will know that they are a dot, dot, dot, dot, dot, whatever, fill in your own blank in there.^7^

Callaghan’s testimony here highlights the legal, societal, religious, and in some cases emotional restrictions on young Irish people’s access to both sexual information and contraception. An examination of the operation of the ACTS thus reveals much about the impact of this landscape on young people, the experiences of activists who were actively trying to change sexual health provision in Ireland, and the intergenerational tensions around this change.

In recent years, the work of teen and young adult-led activist movements such as March for Our Lives and School Strike for Climate has led to greater recognition of the important role of young people in political activism today. As Hava Rachel Gordon has emphasized, this phenomenon reminds us that we should consider young people as ‘political agents in their own right, rather than as citizens-in-the-making who develop into actual political actors and engaged citizens only when they reach adulthood’.^8^ However the longer history of youth activism, with a few important exceptions, has been relatively neglected. In the case of Ireland, while there is a significant emerging scholarship on the history of activism, including valuable studies of feminist and gay activism as well as the activism of organizations involved in the illegal provision of contraceptives in Ireland pre-legalization,^9^ the role of younger people in this area has been ignored.^10^ Similarly, young people, with the exception of valuable recent research by Caroline Rusterholz and Hannah Charnock, have not been the focus of research in the history of sexual and reproductive health more broadly.^11^ The case study explored here sheds new light on the motivations and experiences of the young activists who volunteered with the ACTS. It also explores their activism in the context of their own emotional labour. The significance of emotions in activism and social protest, as both a mobilizing and uniting force, has been investigated by a number of historians, but less attention has been paid to the emotional labour of the activists themselves.^12^ The concept of emotional labour as something which ‘requires one to induce or suppress feeling in order to sustain the outward countenance that produces the proper state of mind in others’ was coined by Arlie Hochschild in 1983, and has since become widely used.^13^ More recently Karissa Patton has highlighted the importance of gender in such labour; her research on the Calgary Birth Control Association ‘epitomises how gendered and undervalued labours of care became woven into feminist health models during the 1970s’.^14^ In the case of operators of a telephone line, as we shall see, we might deem emotional labour to refer to the operators’ withholding of their own emotions and feelings in order to create a safe space for the caller to present their problem.

The 1980s was an unsettling period for many young people internationally. As Gavin Brown and Helen Yaffe have recently demonstrated, young people in the 1980s faced high unemployment, rapid social change, the threat of nuclear war, and the panic of the AIDS crisis, a combination of factors which ‘made many young people fear for the[ir] future’.^15^ Yet the early 1980s were also witness to what has been described as the ‘second youth revolt’ in Europe, characterized by ‘a focus on occupations and militant defence of squatted places, dismissal of political parties and political organisations, a renewed focus on the subjective aspect of politics and a focus on locality’.^16^ Within Europe, youth organizations exchanged ideas, strategies and perspectives.^17^ Moreover, as Daniel Laqua and Nikolaos Papadogiannis have recently argued, young people could gain empowerment from their involvement in internationalist initiatives, and a sense of belonging to a transnational community.^18^

The IFPA was a member of the International Planned Parenthood Federation (IPPF) which enabled the youth group to tap into international networks. In December 1985, IFPA youth group volunteer Jon O’Brien attended a workshop organized by the IPPF Regional Adolescent Services Project in Milan, Italy.^19^ Individuals from family planning groups in other European countries who were considering setting up similar services visited the IFPA to discuss the telephone line and the youth clinic with volunteers.^20^ O’Brien was personally inspired by Elise Ottesen-Jensen, the founder of the Swedish RFSU Group and a founder of IPPF, recalling, ‘I had her on my wall because we very much felt solidarity internationally with people. We felt solidarity with the Poles, you know, living in a Catholic environment.’ Some of the initiatives of the youth group were inspired by projects such as Grapevine in the UK and the Swedish RFSU youth clinics. In O’Brien’s view, ‘that international solidarity was important and inspiring and really gave us energy and kept us going’.^21^

The foundation of the Adolescent Confidential Telephone Service (ACTS) by the IFPA in 1984 followed a longer history of telephone counselling internationally as well as of radical health activism in relation to reproductive rights in the Irish context, particularly around the issue of contraception.^22^ While volunteers on telephone counselling services might not identify as ‘activists’, in Cait McKinney’s words, ‘caring for others using information is vital service work that builds basic movement infrastructures’.^23^ Early crisis counselling hotlines were established in Britain and the United States by religious ministers, such as Chad Varah, a British Anglican vicar who founded the world’s first crisis hotline in 1953 in England (this later became the Samaritans); Reverend Kenneth B. Murphy, a Catholic priest who founded the first American suicide hotline in 1959 in Boston; and Bernard Duncan Mayes, who founded the second in San Francisco in 1961.^24^ As McKinney has shown, ‘hotlines responded to conditions of discontent and the unmet needs of “those who felt alienated by established helping agencies”, including sexual minorities’.^25^ In her history of teletherapy, Hannah Zeavin similarly comments, ‘the phone further allows for an intimate anonymity between peers, where the historical power dynamic and relationship of clinician-patient and priest-parishioner can be left behind for new roles: that of the empowered user and the lay volunteer who can provide something a trained professional can’t, a spontaneous “anonymous ear”’.^26^ In the UK, gay activists were also instrumental in establishing telephone counselling services for the queer community; these included the telephone service Friend founded by the Campaign for Homosexual Equality in 1971, and Gay Switchboard, which was set up in 1974.^27^ Gay and lesbian telephone lines emerged out of the international gay liberation movement of the 1970s, and sought to ‘address isolation from social services, fear of judgement by homophobic hotline operators, and resistance to a counselling psychology that pathologized same-sex desire as mental illness’.^28^

The experiences of individuals using sexual health services can be difficult to uncover due to restrictions on access to client records, and young people’s experiences with sexual health services are particularly difficult to locate in the historical record.^29^ The detailed logbook kept by the IFPA youth group of telephone calls to the ACTS during the course of its existence represents an important window into the key problems facing Irish young people in relation to sexuality and sexual health in the 1980s, and in particular, the experiences of men, who have tended to be excluded from histories of contraception in particular.^30^ I also draw here on the uncatalogued archives of the IFPA, which include press clippings, project reports, memos, as well as newspaper articles, and on oral history interviews I conducted with former members of the IFPA youth group. These interviews were carried out as part of a Wellcome Trust-funded project on the history of contraception in Ireland which entailed 103 interviews with Irish men and women born before 1955, and forty-two interviews with individuals involved in activism from the 1970s to the 1990s. Six interviews were conducted with former members of the IFPA youth group; all except one of these took place in person.^31^

This article makes three key arguments. Firstly, through a close reading of the ACTS logbook, it illustrates the impact of the lack of sexual education and of Ireland’s restrictive laws relating to sexual and reproductive health on Irish teenagers and young people in the 1980s. I argue that the work of the IFPA youth group was not simply an attempt to make contraceptives and sex education more accessible to young people; rather, the young volunteers were motivated by deeper activist aspirations to challenge the conservatism of Irish society and its laws. Secondly, I show that the backlash faced by the IFPA youth group is also emblematic of the inter-generational tensions around sexual health and the modernization of the country and sheds light on broader concerns around young people’s sexuality. Finally, this article also seeks to develop understandings of the emotional labour involved in sexual health activism and show how this was often gendered.

## THE IFPA YOUTH GROUP: ASPIRATIONS AND TENSIONS

The IFPA youth group operated within a strict legal context in relation to access to reproductive healthcare. Contraception was only legalized in Ireland in 1979 with the introduction of the controversial Family Planning Act, described by Charles Haughey, then Minister for Health, as an ‘Irish solution to an Irish problem’. The act had been a disappointment to campaigners both in favour of and against legalization.^32^ It made contraception available for *bona fide* family planning purposes on prescription only; this was widely interpreted as meaning that it was only available to married couples. An amendment to the Act followed in 1985 removing the need for prescriptions for non-medical contraceptives, which could then be sold through licensed premises such as chemists and family planning clinics to individuals over the age of eighteen.^33^ Access to contraception remained restrictive and largely depended on class and location, even after the liberalization of the law; some chemists refused to sell non-medical contraceptives, and not all doctors would prescribe them. Thus in 1985, only 20.5% of Irish chemists stocked condoms. This rose to 45% in 1986, 70% in 1987, and about 80% in 1988.^34^ But even once condoms were legally available for sale from pharmacies they were rarely on display, meaning that their purchase could be a furtive experience.^35^ Without doubt, this was heightened in rural areas and small towns.

Young people were thus particularly affected by both a lack of sex education and a lack of access to contraception, due to legal age restrictions for under-18s, a wider climate of fear and repression around sexual health, and a lack of ‘sympathetic’ doctors and chemists in some areas. A survey conducted by the IFPA in 1983 found that young people were struggling to gain access to sexual health information. The survey revealed that 20% of visitors to IFPA clinics in 1983 were between the ages of seventeen and twenty; among these, 85% had been sexually active for an average of 1.8 years before seeking contraceptive advice, and more than 33% of these had been pregnant in the past.^36^ Access to contraception remained restricted well into the 1990s.^37^ The ACTS therefore was set up in response to the IFPA’s concerns about young people and sexual health and the lack of existing sex education provision in Ireland.^38^ In an interview with *In Dublin* in 1986, volunteers on the ACTS expressed their outrage around the lack of sex education in the country. As Miriam Watchorn, then aged seventeen, stated: ‘There is a very serious problem here in that young people are blamed for getting pregnant, getting VD or whatever, but it’s not their fault if they haven’t been told anything, I think it’s the fault of teachers and parents and society itself. It expects you to know what you haven’t been told.’^39^

The 1983 referendum which introduced the Eighth Amendment into the Irish constitution, thus effectively outlawing abortion, was particularly acrimonious. It came about because of the mobilization of a number of anti-abortion groups such as the Pro-Life Amendment Campaign (PLAC) and the Society for the Protection of Unborn Children (SPUC).^40^ The following year, 1984, Ireland witnessed two distressing cases. The first was the death of Ann Lovett, a fifteen-year-old schoolgirl, who died after giving birth to a stillborn baby at a grotto in Granard, Co. Longford, in January 1984.^41^ The second was the Kerry Babies case, a seventeen-week public inquiry after two new-born babies were found dead within 100 kilometres of each other in April 1984. Joanne Hayes, the mother who had concealed one of the babies, was arrested and charged with murder of the other baby, on the basis of the erroneous claim that she was the mother of both.^42^ As Moira Maguire argues, these two cases were significant ‘because they created a space, for the first time in Irish history, for a wide-scale discussion of the issues that increasingly divided Irish society into “conservative” and “liberal” camps’ – discussion that was facilitated and fuelled by the Irish media.^43^

Yet the period was also witness to radical forms of activism which had their roots in the 1970s, including several telephone counselling services. Members of the Irish queer community played a pivotal role in developing these phone lines; the most prominent was Tel-a-Friend, a counselling and befriending telephone service established by members of the Irish Gay Rights Movement in 1974 and modelled on the London Gay Switchboard.^44^ Other telephone support and befriending services included the Irish Gay Switchboard, housed at the Phoenix Club in Dublin in the 1980s, and Lesbian Line (in Cork, Dublin, Galway, Belfast and Derry in the 1980s).^45^ Members of Ireland’s first pro-choice group, the Women’s Right to Choose Group, established the Irish Pregnancy Counselling Centre (IPCC) in Dublin in June 1980 to provide counselling (and abortion referral if needed) to women facing unplanned pregnancies.^46^ In 1983, following the closure of the IPCC, Ruth Riddick established Open Door Counselling, which included a telephone counselling service called Open Line Counselling. Thus, before the establishment of ACTS, there existed a network of telephone counselling services in Ireland for queer men and women and for individuals facing unplanned pregnancies. ACTS was established, however, out of an acknowledgement that there was a need for support, advice and education relating to sexual health specifically for young people.

IFPA youth volunteers joined the group for a variety of reasons. For most youth group members interviewed, the Ann Lovett and Kerry Babies cases had an important influence on their decision. Miriam Watchorn, who joined the group aged sixteen, reflected on the moral climate of the 1980s in Ireland:

It was terribly repressed, and sex was just a bad word. We’d lived through things like the Ann Lovett case. I don’t know if you’ve heard of that one, but that very strongly affected me because I was the same age as them. And yeah, very, very, very, repressed. Really Catholic, and not a nice place to be for anybody who was slightly different or deviant.^47^

Similarly, John Callaghan, another volunteer, explained:

We all knew how messed up Ireland was. We all knew that the Kerry Babies was happening. There were so many tragedies in the news … There was a palpable sense in the country at the time of anger and outrage and a sense of, ‘This can’t continue. We can’t allow this to happen.’ I can’t remember how old she was [Ann Lovett], she was like 14 or 15 years old. Nobody knew she was pregnant, supposedly. She’s going to a Catholic school with nuns. Conceals this pregnancy right up until the end and then dies giving birth at the foot of the Virgin Mary. The tragedy of that, the ridiculousness of that it gets overwhelming for you, and it’s just, ‘I’ve got to do something. I can’t do much, but I can do that.’^48^

Both Watchorn and Callaghan’s testimonies illustrate the personal impact the Ann Lovett case had on them, and how this, along with emotions of anger and sadness, served as mobilizing forces for them to get involved. As James M. Jasper has argued, ‘anger is a crucial motivation for protest. Without outrage over an injustice, without a villain to blame, there simply is no cause’.^49^ For other volunteers, motivation came from their own personal experiences. Jon O’Brien, then a self-described ‘punk rocker’ from Drimnagh, a working-class area on the southside of Dublin, explained how he noticed that several women he socialized with at the Magnet Bar in Dublin

became pregnant when they didn’t want to be and they either terminated the pregnancies over in England or went on to become single mothers which meant that they weren’t in the bar anymore. Quite a lot of the lads I was with had a real problem getting access to any type of contraception whatsoever. There was a thing going on where one of the lads would, you know, go over to England, buy contraceptives, bring them back, split them up and sell them at cost to other guys in the bar to try to avoid pregnancy.^50^

O’Brien’s testimony here highlights the practical impacts of the lack of access to abortion and contraception on people he knew.

While youth group volunteers foregrounded the sexually repressive environment of 1980s Ireland, their activism clearly instilled in them a sense of hope that they could change Irish society more broadly. As Jon O’Brien stated, ‘I think there was a real sense that some of us really wanted to bring Ireland up to date’.^51^ Similarly, John Callaghan explained:

I was gay. Being a young activist, as they’re called today, just being politically aware, politically active, involved with the Workers’ Party. I grew up in Ballymun. The idea of doing something to change the world and make the world a better place was very much in my head as a younger person. This one just seemed to me, because of my own queerness, an important way to make a contribution and to be relevant somehow.^52^

Such feelings were widely shared. Miriam Watchorn remarked, ‘it was nice to feel that you’re making a contribution and helping in some way. And I would have been at the time, very – quite striving to the idea that Ireland really had to change and grow up and join the twentieth century and get over its Catholic, whatever, inhibitions and things. And there was a sense in which I was helping the cause.’^53^ Siobhán Nowlan’s parents had been involved in the IFPA and it was through them that she was asked to join the youth group, ‘And I thought yeah, let’s make some changes’.^54^ Jon O’Brien reflected on the power of activism to shape politics, stating ‘So we weren’t on the inside, we were on the outside. I think that’s why we went off and pursued sort of alternative strategies, rather than relying on politics to lead us, we had the idea that we would lead politics.’ Moreover, O’Brien explained how the group activities helped him and other members to overcome ‘the certain degree of powerlessness that I think that we felt after the abortion referendum’. He told me:

I think that the whole sort of, you know, ‘Screw you, we’re gonna operate a telephone service, we’re gonna operate a youth clinic, we’re gonna, you know, make information pamphlets, campaigns’, we basically weren’t backing down and we had a very sort of youthful, revolutionary zeal for bringing about real and sustained change in Ireland.^55^

For John Callaghan too, involvement in the youth group represented a means of contributing to social change, particularly in the wake of the Anti-Amendment campaign in 1983, in which he had been involved as a student. He told me:

You lay low, but then you get up and you go in and you sit for four hours and listen to phone calls of people … That, I guess, the good thing about IFPA and the Adolescent Confidential Telephone Service was, while we were losing politically – we couldn’t get the referendums passed, we couldn’t get the laws changed in the way we wanted to – hearing those individual calls and having somebody at the end of the call say, ‘Thanks a million. That’s so great. It’s so wonderful to be able to talk to someone’, that was very uplifting and very rewarding and very positive.^56^

Such accounts show that for members of the youth group, activism offered a means to try and change Irish society by helping young people overcome the stigma surrounding sexual health issues, at a time when the wider political atmosphere around sexual and reproductive health felt oppressive.

In October 1984, the IFPA held a training programme for eleven young women and three young men aged between fifteen and twenty in order to recruit volunteers for the new telephone service.^57^ The ACTS was inspired by youth work in the United States and the role of peer groups in education and information.^58^ During the training sessions volunteers discussed how they felt about their own sexuality and attitudes to sex, before undergoing training in counselling callers on the phone.^59^ Volunteers self-selected whether they would remain on the programme after training; following the completion of the training in October 1984, eight of the original fourteen members began the pilot project.^60^ Jon O’Brien, one of the original volunteers, was employed as Youth Officer from 1985 and given responsibility for the youth group. Following the success of the ACTS pilot project, the service was launched in January 1985.^61^ Ongoing training was provided as part of the project as the group developed over the years.^62^ The telephone service was funded by the IFPA, as numerous national funding grant applications were unsuccessful; O’Brien reported in 1987 that he believed this was because ‘our work on sexuality is still a sensitive and controversial subject in Irish society’.^63^

Volunteers were very young and, in some cases, sexually inexperienced. Finn van Gelderen, for example, commented, ‘I think about it now, I hadn’t actually ever had sex and I was on the telephone talking to people about sex, and I thought that was a bit mad.’^64^ Telephone Answering Technique Training (TATT) was developed by O’Brien and the youth group in 1985 and utilized at subsequent training sessions. This involved the volunteers exploring the key types of calls received by the service and ‘compiling detailed information on the responses required and finally putting a structure to these answers’; they were encouraged to use ‘the language of the street’. They then practised asking and answering questions with each other. Volunteers also explored how to deal with abusive and challenging phone calls, and after each session there was a debrief for volunteers to process challenging calls, as a way of collectively learning how to deal with such calls in future.^65^ An information directory, organized A to Z by subject (e.g. ‘pregnancy’), was produced by Jon O’Brien to aid youth volunteers on the phone line, and this was regularly updated by the volunteers over time.^66^ The TATT illustrates efforts at professionalism, perhaps responding to the ages of the young volunteers, as well as attempts to create an environment of openness and space in which the callers could be heard.

Volunteers were primarily encouraged to offer support and reassurance to callers. According to Siobhán Nowlan: ‘We wouldn’t guide them, we would listen in a way that they came to their own rightful decision’.^67^ For cases of rape and incest, for instance, volunteers were advised to tell the caller about the Rape Crisis Centre and what to expect, ‘Tell them it was not their fault/they are not alone. It’s their choice to go further. Spend time with them; support, advice; reassurance’, and also, ‘it is very important that you – the volunteer – talk about the call after with partner’.^68^ Resources used in training included discussion cards produced by the British Brook Advisory Centres. The group also drew on techniques used by the Samaritan telephone service, ‘concerning caller centre response, encouraging callers to talk, reflecting strong emotions etc.’^69^ Ultimately, the ACTS represented an attempt to create a new way of talking and thinking about sexual health in Ireland which was inspired by international approaches. In Callaghan’s view:

IFPA and I think it was Christine [Donaghy] largely, Jon O’Brien too, took that risk and empowered us and trained us and encouraged us, with this sense that you’re changing individual people’s lives and you’re changing the culture at large. We knew it was really important. We knew it was a big deal. We were motivated by both the immediate needs of that person and the fact that you were pushing the culture and pushing the society along. We all knew that, and we were there to do it.^70^

While the youth group volunteers had a clear sense of what they wanted to achieve through their activism, their work was controversial, and sparked criticism from conservative groups, politicians and members of the public. Such disapproval was not distinct to Ireland; in the UK, there had been much controversy over the establishment of the Brook advisory clinics in the 1960s, amid concerns that they would promote promiscuity among young people.^71^ Fine Gael party TD Alice Glenn, for instance, in 1985 referred to the ACTS as a ‘sex hotline’.^72^ The ACTS volunteers also found themselves in a position of having to defend their work in the face of backlash. In February 1985, a call from the *Sunday Tribune* asked ‘what we thought of Alice Glenn’s comment that this organisation is a sex hotline & that it is undermining the family. Told him info was wrong & he asked about failure rate in condoms. Told him 5–10%.’^73^ Irish agony aunt Angela Macnamara also criticized the service in a letter to the *Sunday Independent* in December 1984, drawing attention to the IFPA’s links with the International Planned Parenthood Federation, and encouraging readers to ‘try to stop this service or if not just phone them and see for yourself what their main thrust might be.’^74^

Criticisms of the IFPA youth group and the ACTS also reflected wider concerns from conservative groups about changes in society, young people’s sexuality, and the engagement of international groups in Irish moral issues. In 1984 the Irish branch of the Responsible Society, a British morally conservative group, condemned the ACTS. They stated in their newsletter that ‘the people manning the phones were young untrained and unemployed people’ and noted that the service ‘covered *pregnancy counselling* [their italics] and contraceptive advice’.^75^ Additionally, the group asserted that the ‘whole sordid scheme’ stemmed from influences outside of Ireland, such as the International Planned Parenthood Federation.^76^ And in 1985 they argued that ‘the purpose of such youth counselling services is to motivate young people to use contraceptives’, claiming that such services promoted promiscuity and that there was a ‘high failure rate of condoms among adolescents’.^77^ In a more extreme expression of hostility, when the youth group went on to establish a Young People’s Family Planning Centre in 1986 where they provided in-person sexual health advice, the IFPA received a bomb threat on the eve of the launch of the centre.^78^

The creation of the telephone line and the broader changes sought by the volunteers were not without tensions within the IFPA either. For Miriam Watchorn, staff concerns were twofold: there were apprehensions firstly that the youth group was a drain on resources, and secondly, that the members were too young to be involved in the provision of sexual health advice. She explained: ‘Maybe just our youth. Maybe we were too young for some people. We were underage. I was certainly under the legal age for sex at the time I got involved.’^79^ Moreover, the societal change and the new approaches to sexual health that the volunteers were seeking were unsettling for older members of the organization. According to John Callaghan, ‘I think that some professionals, nurses, counsellors, psychotherapists, social workers might say, “Are you sure you want a 20-year-old, 19-year-old talking about abortion, talking about contraception? What if there’s a plant in there? What if some journalist or one of the opposition groups get you saying, making mistakes, or are these young people mature enough to handle what’s happening?”’^80^ Jon O’Brien similarly recalled, ‘It was very new, the idea of young people being involved in service provision, and we had to work very, very hard to gain acceptance. And I think if it wasn’t for people like Christine Donaghy the whole thing wouldn’t have got off the ground, because some of the older people really had a problem with it.’^81^

The move to a more person-centred approach to sexual health counselling, as opposed to the medical model which had been central to the earlier history of the IFPA, also created intergenerational tensions. As O’Brien explained in a 1990 project report reflecting on the IFPA youth group, because the IFPA had had a strong medical model since its inception, ‘The idea of bringing in non-medical young people, training them and working with them was a difficult transition process and it required time and patience to gain acceptance and support among our membership’.^82^ When the ACTS was first established, many staff in the IFPA were concerned that young volunteers would not possess the skillset to answer questions and provide advice on the telephone. In order to offset these concerns, older IFPA staff members volunteered to supervise the young people; however, ‘after a few months, it became apparent that there was no need for this supervision to continue, especially when a supervising top gynaecologist admitted that he did not know the answers to some of the questions but that the young volunteers did’.^83^ Similarly, following the establishment of the Young People’s Family Planning Centre, an executive committee member wrote to the youth group to complain about the provision of free pregnancy testing, ‘as young people may rely on preg. tests rather than use contraceptives’. She also pointed out that there should not be a discount price for contraception for young people because ‘cut pricing contraceptives is bringing “making love” into a Quinnsworth [Irish supermarket chain] arena and therefore cheapening it.’^84^ Other staff shared concerns about the involvement of youth volunteers in the operation of the service. A staff meeting was held to allay these fears; however, the group found ‘that for the duration of the project new fears and reservations arose on a regular basis’, a problem believed to have more to do with ‘the culture and structure of the Association rather than a failing on behalf of those in charge of the project’.^85^

Ultimately, it is evident that volunteers were motivated by a combination of personal experiences, tragedies in 1980s Ireland, and wider aspirations to change attitudes towards sexual health provision and Irish society more broadly. Acknowledging the prejudices around their relatively young age, volunteers worked to create a professional service through the use of rigorous training and resources. Yet the concerns expressed not only by conservative members of the public but also by older members in the IFPA around their activities highlight the intergenerational tensions around the type of change they were aspiring to create, and how controversial this work was in the Irish setting.

## CALLS TO THE ADOLESCENT CONFIDENTIAL TELEPHONE SERVICE

**Figure 2.**
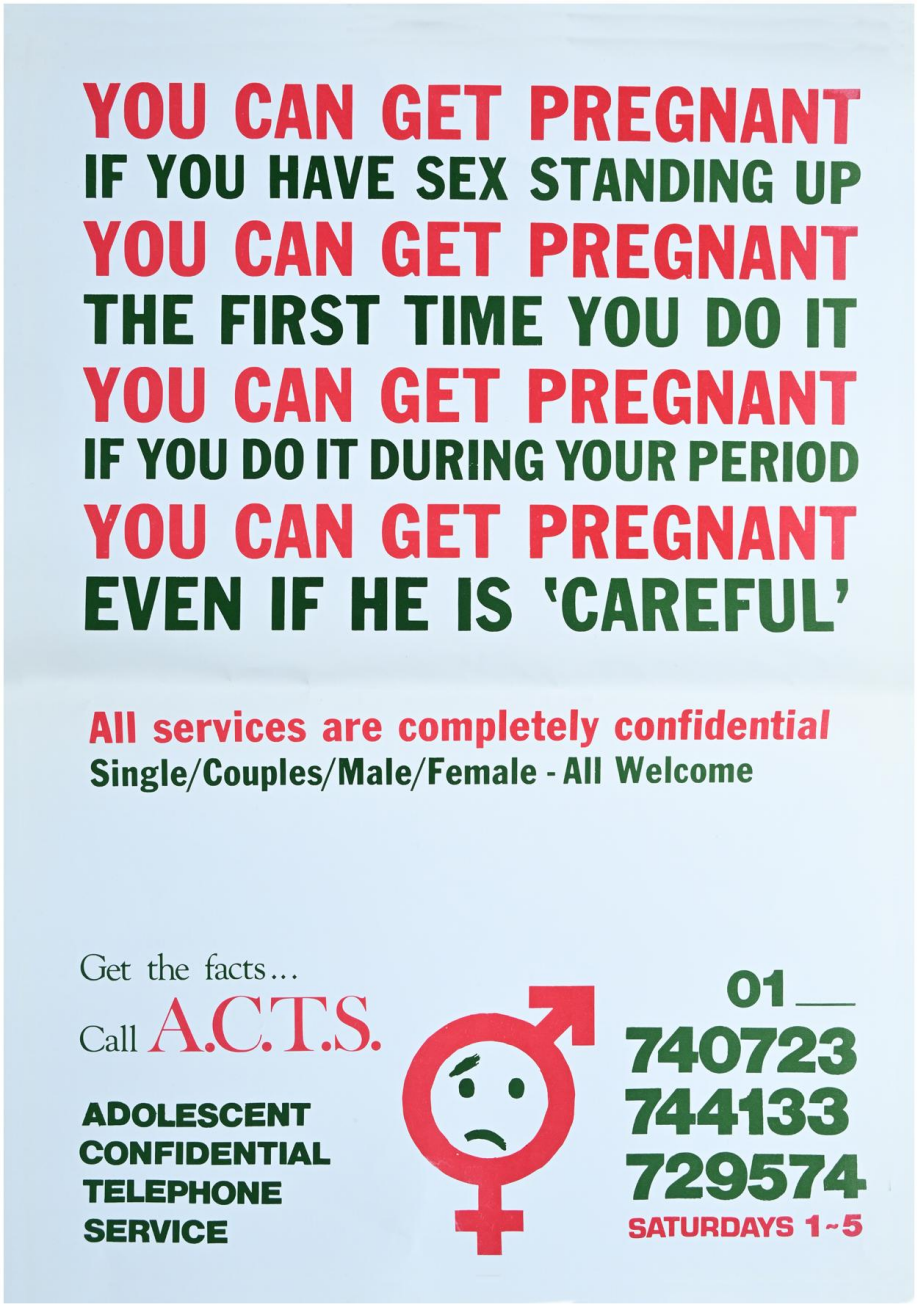
ACTS poster, 1986. Irish Family Planning Association archive, RCPI Heritage Centre, Dublin. With thanks to Harriet Wheelock.

A closer examination of the nature of the phone calls received by the ACTS allows for analysis of what they reveal about Irish society and young Irish people’s experiences of sexual health in the 1980s (Figure 2). Like many other telephone counselling services, the individuals staffing the ACTS kept a detailed logbook outlining the calls received to the service and the advice given. The logbook enabled the youth group to compile statistics relating to the service.

Importantly, young men represented the majority of callers to the telephone line. This might suggest that our tendency as historians to look at contraception as a ‘woman’s issue’ is restrictive; the logbooks are a rare window into men’s agency around this issue. In the first year of the telephone service 80% of callers were male and 20% were female, and this preponderance of male callers continued throughout the lifetime of the project.^86^ Male callers made up 79% of callers in 1986, 74.8% of callers in 1987, 87% in 1988, and 85% in 1989.^87^ There are a number of possible explanations for this pattern. It could suggest that young men were active agents in seeking information around sexual and reproductive health, or that young men were particularly affected by the country’s sexually repressive environment and lack of information. Alternatively, the logbook entries might indicate that it was often men who took responsibility for a sexual health issue within a relationship; for example, there are numerous entries where the male partner phoned the line to ask for support regarding an unplanned pregnancy. It may also suggest that women were more affected by embarrassment and shame, and hence reluctant to phone the telephone line, as well as being more acutely concerned about confidentiality, particularly in the case of an unplanned pregnancy.

Moreover, the telephone logs offer a glimpse into the dynamics of individuals’ personal relationships. In April 1988, for instance, a phone call from a male in his twenties was logged as follows: ‘Wanted to know how a girl gets pregnant. Told him all about conception and contraception. He then told me he was having sex with his girlfriend and wasn’t taking precautions. Recommended use of condoms/pill.’^88^ Similarly, a call from a male teenager in June 1989, was logged as, ‘Had started having sex with girlfriend – was worried about her getting pregnant. He didn’t like using condoms & she felt embarrassed about going to doctor for pill. Told him he would need to use something & using condoms would be better than her getting pregnant.’^89^ These two calls illustrate the impact of Ireland’s legislation regarding contraception on individuals’ relationships and the risk-taking that often ensued. They also complicate the picture in relation to responsibility for family planning in relationships. In the case of earlier generations of Irish people, it appears to have been predominantly women who took responsibility for family planning in heterosexual relationships, yet the logbook entries suggest that young men coming of age in the 1980s were taking more responsibility for this issue.^90^

Over forty-three weeks of operation in 1986, 653 calls were logged, an average of fifteen calls per Saturday afternoon. Most callers were young people; 37% were aged 16–20 and 29% aged 21–25, while 17% were aged 26–30.^91^ At the launch of the project report in 1986, O’Brien commented, ‘We were surprised at how basic the questions were. We had callers over 20, for example, asking about pubertal changes.’ O’Brien added, ‘The response to this service has emphasised the need for comprehensive sex education not only in schools but also through Youth Clubs throughout the country’.^92^ While most calls came from individuals in their teens and early twenties, there were numerous calls logged from older married men and women asking for advice, showing that older people were also lacking information and availing themselves of the service. In an interview for *In Dublin* in 1986, Jon O’Brien summarized callers to the service as, ‘People who had suffered for years in ignorance, who had sexual problems about which they could get little if no information. People who belonged in a sense to the rigid “macho” world of men who couldn’t dare admit to having any sexual problem at all.’^93^

Calls concerned a huge variety of issues, which volunteer Gerry Curran summed up as follows:

They’d ring up, it could be, ‘Where can I buy condoms?’ Or it could be, ‘I think I’m gay.’ Or it could be, ‘I think I’m pregnant.’ Or it could be, ‘I was raped last night.’ Or it could be, ‘I have a disease.’ Or it could be, ‘I get turned on a bit by wearing women’s clothes, is that all right?’^94^

The most common calls were those requesting information on contraception (representing 15–20% across the five years), STDs and AIDS (10–20% in the first three years of the service, with calls relating to AIDS peaking at 12.9% in 1987 following increased media attention, then dropping to 6–7% in 1988 and 1989). Calls relating to relationship problems, sexual problems, sex education, and masturbation were also common.^95^ The records of these calls illustrate the range of issues that callers had. Logbook entries highlight a lack of knowledge among many young people who phoned the service. For instance, in February 1986, the logbook recorded details of a call from a 16-year-old male from Dublin, ‘enquiring if a girl could become pregnant through oral sex or by masturbating. Told about contact pregnancies and condoms. Asked if there was an age limit, answered he would get condoms in Cathal Brugha St. [Family Planning Clinic]. He was delighted.’^96^ The relief of the caller is palpable from this entry.

Calls to the phone line highlighted the fact that, as Jon O’Brien commented in a 1987 newspaper interview, ‘many young people have a contradictory attitude to sex – namely it’s all right to do it, but not all right to plan to do it, and take precautions accordingly’.^97^ For example, a 22-year-old male caller to the line in January 1986 explained that he was having intercourse with his girlfriend without using contraception and that his girlfriend had recently had a pregnancy scare. He was ‘told re: methods of contraception and clinics. Advised to go to FP Clinic with girlfriend to see doctor.’^98^ Calls did not just come from people asking for information or sexual health advice; the logbook also records a number of calls from teachers wanting information on how to better educate their students, parents looking for advice in relation to their children, and in one case, a doctor asking for information on where to buy an IUD.^99^ Such calls reflect the paucity of basic information about sexuality and sexual health in Ireland among all age groups, and show how the ACTS began to fulfil a wider role than just sexual health counselling.

The logbooks also provide information about the regional distribution of helpline users. The majority (57%) of callers in 1985 came from the Dublin area, with 34.1% coming from outside of Dublin, and 8.9% from unknown areas.^100^ The higher percentage of callers from Dublin reflects the fact that 26.5% of the population of Ireland lived in Dublin at this time; additionally, the telephone line was more effectively advertised in the Dublin region through the *In Dublin* magazine.^101^ Callers in rural areas were seen as a particularly vulnerable group, and it is clear that the death of Ann Lovett cast a long shadow. The ACTS volunteers often dealt with callers who were fearful and upset. In an interview for *In Dublin* in 1986, O’Brien stated, ‘The most harrowing calls are those we get from rural parts of Ireland, from pregnant teenagers. They are emotional, panicky and very frightened. Potential Anne [sic] Lovetts. Our job is to get them in contact immediately with a doctor in their area. If there isn’t one, it makes it all the more frightening for the teenager in question.’^102^ One such call, logged in February 1985, recorded, ‘Girl (Sligo) pregnancy testing. Afraid of clinics and doctors in her area. Ref to Sligo F.P. doctors and Dublin clinics.’^103^ This entry indicates the concerns of young people over approaching clinics and doctors in their local area for fear that their confidentiality might be broken.

Other calls from the logbook highlight the regional disparities in accessing contraception. For example, one caller in January 1985 from Clare, ‘wanted to know about contraception. Himself & girlfriend both 16. Told him no Family Planning Centre in Clare, so would have to try doctor’.^104^ Consulting a doctor about contraception in a rural area may have been challenging for some individuals, for fear that the doctor would tell their parents, or that they would be seen in the local chemist obtaining the prescription. The IFPA therefore compiled a list of ‘sympathetic’ doctors who would be willing to provide family planning services in their area.^105^ Volunteers on the phone line were also advised to refer callers to the IFPA clinics where necessary, and for callers based in rural areas, ‘refer nearest clinic or country doctor list – Red dot – doctors we know’.^106^ For instance, a 35-year-old male caller who rang the line in March 1988 with a query about methods of contraception was ‘referred to ‘Red Dot’ doctor in Kildare for a talk’.^107^ The calls also shed light on the practical challenges facing young people who wanted to gain information on sexual health issues, such as a call in October 1985 from a male aged 17–20 which was ‘interrupted by somebody entering his room’, while another caller in December 1984 ‘hung up/rung off before he had a chance to ask more. Phone was asking for money “Beep, Beep, Beep”’.^108^

The logbook also sheds light on the gendered dynamics of telephone counselling. There were many instances where male callers phoned the service and asked to speak to a female counsellor. Recalling his work on the ACTS, Finn van Gelderen stated, ‘I remember that out of I’d say 95% of the calls that I got, people hung up on me, because I was a man.’^109^ The logbook entries underline this: for a session in February 1987 it was noted that that ‘8 callers wanted to speak to female – referred to next Saturday’.^110^ According to Jon O’Brien, in a 1986 interview, there were two reasons for why callers might ask to speak to a female volunteer. First, he suggested, ‘I suppose men are supposed to know about sex “naturally”. They don’t often like to admit to another man that they have a problem.’ O’Brien also alluded to wider societal stereotypes around women’s natures, stating, ‘I think also many men would believe women are more compassionate. It’s not as bad for them to admit their inadequacies to women and there is the female image there for men too. Women tend to talk more openly with each other about sex and their bodies.’^111^ There was just one instance in the logbook, recorded in July 1988, where ‘Fella wanted to talk to a man, told him to phone next Saturday or during the week.’^112^

There was also a wider fear among some callers about confidentiality, or that their parents might find out about the issues they were having. For example, a male caller in November 1984 ‘thought he had V.D., he explained symptoms, was worried about going to Doctor because he thought the parents might find out. I said they wouldn’t find out. He then hung up’.^113^ This entry sheds light on the concerns of young people around asking sexual health services confidentially, without their parents being informed. In particular, when women rang seeking information on abortion, according to John Callaghan, ‘they almost never spoke to men, so as the male on the line I would get hung up on. We would kind of know, “Okay, I’m not going to pick up the phone for the next 30 minutes in case this person wants to call back.” They would talk to women more freely about “I’m in trouble. I need help getting…how do I do this? How much does it cost? Will people know? Do I have to give my name?”’^114^

The ACTS logbook also illuminates the challenges facing women experiencing unplanned pregnancies in 1980s Ireland, experiences which can be challenging for historians to uncover.^115^ The logbook contains many entries from women who were fearful of an unplanned pregnancy and seeking advice. It also provides a glimpse into the fear, trauma and anguish experienced by women in this period without access to information on how to deal with an unplanned pregnancy, or access to legal abortion. For example, a distressed 25-year-old female caller from Dublin phoned the ACTS in August 1985. The call was recorded as ‘Female thought she was pregnant – condom burst he hadn’t come yet though. Period was three days late’. Later that afternoon, the same woman called back and ‘asked about taking a bottle of gin & a hot bath. Advised her against it. Told her not to worry. (She was married with 2 children).’^116^ These two phone calls highlight the panic facing the caller in facing a potential unplanned pregnancy, and again, the potential recourse to a self-managed abortion because abortion was illegal in the country. It also re-emphasizes that it was not just young, single people utilizing the phone service. Other calls shed further light on the challenges facing Irish women who sought or had abortions in England in the 1980s. A 21-year-old woman from Dublin, for example, rang the ACTS in February 1988:

Wanted abortion referral – 21 weeks pregnant. Spent 4 days in London looking for abortion – was to come back each day for 4 days – eventually told the clinic was booked up for over a week -> would have to come back. Referred to IFPA clinic in hope of help.^117^

The practical and emotional impacts of the Eighth Amendment, and the wider climate of fear around attempts at finding basic information on abortion, are clear from the records of these calls from young women. More broadly, as this section has sought to show, it was not just young people who were affected by the legal, religious and societal restrictions relating to sexual and reproductive health. The ACTS offered individuals, particularly young men, a way of openly accessing information and being listened to in a way that fought against a wider culture in Irish society that insisted on secrecy, shame and silence around sexuality and sexual health.

## EMOTIONAL LABOUR, CHALLENGES AND BACKLASH

As well as providing insights into the nature of calls to the service and the key problems affecting young Irish men and women, the ACTS logbook also provides insights into the significant emotional labour and empathy of the activists, as well as the practical, and often gendered, challenges of doing this work. Occasionally the young volunteers had to deal with difficult calls concerning rape and incest. For instance, in February 1985, the logbook recorded a 19-year-old male caller from a rural part of Ireland who explained that he was being sexually abused by his 25-year-old brother. The volunteer ‘referred him to the rape crisis centre. His brother has moved out of the house now but was sleeping in the same bed as him. He now finds it hard to live with what was done to him’.^118^ Similarly, in May 1986, Miriam logged a phone call as follows:

Girl had been raped. Extremely upset. Tried to reassure her & suggested R.C.C. [Rape Crisis Centre]. Told her that they were the best people to help her – they would understand what she’s going through – would provide doctor etc. confirmed that counsellor was in RCC and persuaded her to ring. I hope she did.^119^

This call had a significant impact on Miriam, and our oral history interview enabled her to discuss the emotional impact of the work on the phoneline, something which is only hinted at in the logbooks. In the course of our interview, she mentioned, ‘I did get a rape call once and that was very … that was quite, yeah, it was hard to deal with’. Later in our interview, Miriam returned to this incident, stating, ‘I wasn’t concerned for myself, but I was concerned that she would get the proper help and attention she needed. And hoped that she would ring the numbers or the number I gave her.’^120^

Another call logged in November 1986, which was highlighted in pink by the volunteer, illustrates the emotional labour involved particularly for female volunteers. A 17-year-old male caller from Dublin had phoned the line and asked to speak to a female volunteer that he had spoken to previously. This was logged as, ‘Siobhán: this guy keeps on calling wanting to talk to you. He said he was following your advice and it was good so far. Next time you talk to him I would advise you to point out that it is not on for him to keep on calling you otherwise he will become dependent on you.’^121^ The following week, another call was received from the same person, logged by another volunteer as ‘Siobhán’s friend rang – told him to make his own mind up – not to become dependent on service.’^122^ Occasionally callers would follow up to let the volunteers know how they had got on with the advice. For example, in March 1990, a call was logged as ‘Message for ‘Robert’, i.e. John C. – ‘It’s back to normal again’ – I think that this refers to call no. 9 from last week, i.e. that the guy that thought he had an STD doesn’t!’^123^

As well as dealing with emotionally challenging phone calls, the ACTS volunteers also, in the words of Finn van Gelderen, ‘slowly discovered that actually some people were using this line for certain purposes, which were not exactly what it was designed for.’^124^ Alongside genuine calls searching for support and help, the logbooks detail many calls where the caller was masturbating on the phone, in addition to ‘hang-ups’, hoax calls, potential attempts at entrapment, and calls from individuals who fundamentally disagreed with the existence of the service. Such calls illustrate the challenges which were particularly faced by female volunteers on the phone line and the vulnerable position they were placed in through doing this work.^125^ For example, a call logged by a female volunteer in January 1985, recorded, ‘Male masturbating dressing up in women’s clothes, worried, gave him no. of psycho-sexual clinic and said I preferred if he didn’t masturbate over the phone.’^126^ A call logged by a female volunteer in April 1985 from a 50-year-old male caller was described as ‘Had marital problems. Was lonely. Asked me if I would have sex with him.’ Similarly, another call logged in May 1985, was described as ‘Very confused person wanted to talk about sex & exciting positions, he got very excited and vulgar at stages but when I said I prefer if he didn’t masturbate he apologized & talked on’.^127^ As stated earlier, volunteers held regular debrief sessions in order to process such calls. As well as the in-person debriefs, the logbook enabled volunteers to air their frustrations regarding such calls and communicate with each other about these. For example, in August 1985, a call logged by a female volunteer from a man in his twenties, stated, ‘Man wanted to know how to masturbate to get maximum satisfaction. Told him there was no set way, just whatever suits you best.’ The volunteer wrote beside this ‘(how come I always get these calls?)’, and another female volunteer, Lesley, responded ‘(I get all the rest of them. Lesley.)’^128^ Similarly, in September 1987, Lesley recorded the following call from a male caller aged twenty, in the logbook: ‘Another bloody wanker!! Masturbates 3/4 times a day. Does everybody do it? How do married people do it?? (This guy is weird). I got a full description of how he masturbates (great fun). He is living with his aunt. Should he tell her he masturbates & should he stop.’^129^ Clearly, for these young female volunteers, the work on the phone line could at times be personally challenging and frustrating. The harassment from male callers in the above examples again emphasizes how the emotional labour of the phone line was gendered, with female volunteers having to deal with issues that male volunteers did not.

There were also hoax calls made to the service. For example, in March 1985, a series of prank calls by a young male caller were recorded. In his entry describing these in the logbook, Jon O’Brien noted, ‘This is the guy who is the crank who phones every week I talked to him about it.’^130^ Similarly, a series of hoax calls during a session in August 1986 were recorded as following:

3.35. “What is masturbation?” This is the little shithead who rings all the time.

4.02. Shithead again – this time “What is oral sex”. Told him and then said goodbye. M, Dublin 16.

4.20. Shithead again – he asked what making love is. I said “I think you rang before”. He said he hadn’t. I said I recognised his voice and he had rang twice already today – he hung up.^131^

This entry clearly illustrates both the patience and frustration of the volunteer and the importance of the logbook as a space to record negative emotions that were necessary to withhold on the phone. As Cait McKinney has suggested, ‘By descriptively logging emotionally challenging calls, volunteers might imagine others on subsequent shifts helping them to hold these difficult conversations. Reading in the margins of call-log sheets, it seems that volunteers needed places to put all the bad feelings they weren’t going to reveal to callers.’^132^

Moreover, the young volunteers often had to deal with backlash or abusive phone calls directly on the phone, and the group also received hate mail. As Finn van Gelderen remembered,

I had somebody ring me up once and tell me I was the spawn of the devil and all this kind of stuff and that how immoral we were and that we were promoting contraception and sure, that’s the work of the devil and all this kind of stuff, and we just very calmly said ‘I’m terribly sorry you feel that way’ and got them off the phone as quickly as possible.^133^

Van Gelderen’s clear recollection of this incident, almost forty years later, underlines its emotional impact. Similarly, Miriam Watchorn recalled, ‘We got a few abusive calls as well. We had the odd person ringing you up and telling you you’re going to hell in a handcart and this kind of thing.’^134^ The ACTS phone log also records some hostile calls. For instance, in February 1985: ‘Woman phoned to complain about article in Irish Times newspaper on phone service. Said it was the right of the family to teach sex-ed and no phone service could do it. Also said Sex ed was best taught by watching cows, cats & dogs in street!’^135^ Similarly, in March 1985, Miriam recorded a call as follows: ‘Woman criticising whole set-up. Questioning me on my views. Very antagonistic.’^136^ Such calls again reflect the intergenerational tensions around young people providing sexual health services, as well as growing fears about young people and sexuality.

Given the moral climate, volunteers were wary about being monitored by conservative groups. In a 1985 article on the telephone service, IFPA doctor Mary Short explained, ‘You have to be very careful what you say. You don’t know who you might be talking to.’^137^ Fears of entrapment were of particular concern to the youth group following an injunction granted to SPUC by the High Court in December 1986 against two pregnancy counselling services run by Open Door Counselling and the Dublin Well Woman Centre. As a result, Open Door Counselling was forced to shut down, and the Dublin Well Woman Centre had to suspend its counselling services.^138^ The ACTS received many phone calls relating to unplanned pregnancy, and according to Siobhán Nowlan, ‘But obviously with everything being illegal, you had to be very careful what you said. And often we’d say, go visit the clinic.’^139^ These callers were usually referred to an IFPA clinic for advice, or, prior to the High Court injunction in 1986, to the Well Woman Centre or Open Door Counselling. For example, a woman calling the ACTS in 1987 was logged as, ‘Thought she might be pregnant, period 18 days overdue. Referred to Limerick F.P. clinic for test. Talked to her for a while as she was very worried.’^140^ Another caller in 1989:

Had an abortion 3 weeks ago. Crying on the phone. Wouldn’t talk much. Didn’t want to go for counselling in case they arrested her. Referred her to Well Woman. 20s, F, Dublin.^141^

This log not only illustrates the caller’s distress on the call but the lonely position facing women returning after having an abortion in England, and their fears about the legality of their choice, particularly in the wake of the referendum on the Eighth Amendment and the recent shutting down of abortion referral services and information in the country.

The logbook also records numerous calls relating to abortion or unplanned pregnancy where the volunteers suspected that they were being set up. For example a call recorded by Lesley Smyth in 1986, supposedly from a 25-year-old woman in Dublin, was described as ‘Wanted info on termination – sounded a bit iffy so I referred to Well Woman – sounded as if we were being sussed out.’^142^ Similarly, in January 1987, the logbook recorded the following call taken by Jon O’Brien, supposedly from a 45-year-old woman from the country: ‘was married with 4 children, wanted to know about abortion. This was a set up. I kept within high court guidelines.’^143^ Again in April 1988, the logbook recorded ‘Saw our Ad wanted to know about 1) V.D. 2) Conception 3) Abortion. Read from book. I thought it might have been set up.’^144^ And in February 1990, a young man who claimed to be seventeen phoned asking about how to obtain condoms; the volunteer who took the call wrote ‘Sounded older so wasn’t sure if it was someone checking us out.’^145^ Such set-up calls suggest the callers assumed that because of the young ages of the volunteers, they might be more susceptible to being caught out; however, in many cases, as a result of their experience, the volunteers felt able to detect whether a call was genuine or not.

For many of the IFPA youth group volunteers the work was empowering, and it led some volunteers into careers in the arena of sexual health activism. Siobhán Nowlan went on to become a family planning and sexual health nurse in England. Jon O’Brien went to London to work for the International Planned Parenthood Federation in 1992, after which he returned to the IFPA on secondment before going on to help set up family planning associations in Estonia, Latvia and Lithuania as well as helping with the reform of associations in Poland, Hungary and Cyprus. He also worked as press officer for the 1992 Irish campaign in support of the successful referendum on freedom to travel for an abortion and freedom of information on abortion. He later became president of the pro-choice organization Catholics for Choice. Other members went into disparate careers, but each recognised the significant role the youth group had played in their lives, and the important friendships formed from it. John Callaghan, who moved to the U.S. in 1994, stated that he continued ‘in, around left-wing politics, but not in a terribly active way’, but that the youth group ‘was very important for me. It was great work. I loved it. I made many enduring friendships out of it, but it didn’t become a vocation for me or a lifetime commitment.’^146^

While the work was emotionally challenging, it is evident that the youth group volunteers also found it rewarding. There were many calls logged where the volunteers recorded the positive feedback received and the palpable relief and gratitude of callers. For example, a call received in May 1986 where the person ‘wanted to know about contraceptives and about different books’, stated ‘He said he thought the service was a really great thing’.^147^ Another call on the same day was logged as ‘masturbation is bad & harmful – felt guilty. Said we helped a lot.’^148^ Similarly, a call from a twenty-two-year-old man from Dublin, who believed he was gay, was logged as, ‘He was v. glad to ring as I was the first person he told.’^149^ As John Callaghan explained, ‘The thanks you would get from them was so sincere and so palpable, it was a huge reward just knowing that you had actually made a difference in their lives, really, really. I know that sounds schmaltzy but it’s so real.’^150^ Reflecting on the positive impact of this work, Gerry Curran commented, ‘I liked it. I liked them ringing. It was very worthwhile. Particularly if you listen to someone who goes, “Oh thanks, you’ve taken the worry off my shoulders.”’^151^ As Cait McKinney has argued in her study of the Lesbian Switchboard, ‘the caring practices that transpire when a phone is answered are singular, even minor in a larger struggle, but they make social movement participation and service work matter in the everyday lives of individuals’.^152^ While the ACTS provided an important service to young people in this period, it also provided meaning for its volunteers, and enabled them to be feel part of radical activist attempts to change Irish society, and particularly attitudes around sexual health. Their experiences echo Deborah Gould’s point that ‘a movement’s demonstrations, actions, and other events – its rituals – allow participants to move outside of the everyday mundane […] out of everyday devastation, and to be transported into a more meaningful existence that holds out potential for self and social change’.^153^

## CONCLUSION

The telephone calls recorded by the ACTS volunteers in the mid to late 1980s offer a unique window into the impact of Ireland’s legal and religious restrictions. As soon became clear, it was not just the target client base of young people who made use of the service; the calls revealed the chilling impact of the broader sexually repressive moral climate on older people too. The fact that men comprised the majority of callers to the phoneline, as discussed above, suggests that they were particularly affected by this climate, or perhaps that they were more likely, in the case of heterosexual relationships, to be the partner who sought advice. The controversy and backlash in relation to the work of the IFPA youth group is indicative of wider tensions around the modernization of Ireland, and around young people’s sexuality, in the wake of the legalization of contraception in 1979 and the death of Ann Lovett in 1984. Members of the youth group effectively confronted three challenges: young people and sexuality, the power dynamics within health organizations, and young people’s involvement in social change. The young volunteers on the telephone line not only had to deal with the task of providing information to those who needed advice, but also had to learn to manage demanding situations including hostile calls, the emotional labour of responding to distressed callers, and potential entrapment. Reading between the lines of the calls logged by the ACTS, the fear, worry, anguish, shame and embarrassment of the callers are ever-present. Yet it is clear that this phone line, operated by young, and in some cases sexually inexperienced, volunteers, also had a positive effect for callers, empowering them to take responsibility for their sexual and reproductive health and providing them with reassurance, resources and support in relation to a myriad of issues.

While historians have explored the emotions of activism, there has been less focus on the emotional labour of activist work. This case study of the IFPA youth group illustrates both the power of emotional labour in social movements and also the impact of this labour on activists; I encourage scholars of activism to pay further attention to emotional labour as a form of radical activism as well as considering how this was often gendered. Moreover, we should expand our definitions of what activism means; while former volunteers might not have described themselves as ‘activists’, it is clear that their work was powerful in terms of its impact for clients and in generating wider discussion around sexuality and sexual health in Irish society. The IFPA youth group activists were empowered by this work and by a sense that they were helping to change Irish culture and attitudes.

Indeed, by the time of the disbandment of the group in 1990, this cultural change was on the horizon. In 1993, the Health (Family Planning) Amendment Act removed the restrictions on where condoms could be sold and the minimum age requirement for those buying them, and in the same year homosexual acts were decriminalized. In 2015, same-sex marriage was legalized after a referendum. In 2018, the Eighth Amendment of the constitution was repealed, also by popular vote; abortion is now legal up until twelve weeks of pregnancy, as well as after twelve weeks in cases of fatal foetal abnormality or where the pregnant person’s life or health is at risk. Nonetheless, recent research by Grimes and others has shown that there are still significant barriers in terms of access after twelve weeks.^154^ In addition, individuals seeking an abortion have to undertake a statutory three-day waiting period between their first consultation and having the abortion, necessitating at least two visits and sometimes leading to delays.^155^ More broadly, the issue of sex education remained controversial well into the 1990s. And while the introduction of free contraception since 2022 is a positive move, its restriction to female forms of contraception for those aged between seventeen and thirty-five reminds us of the persistence of anxieties around young people’s sexuality which, along with tensions over sexual health more generally, have continued to characterize recent legislation.

## Funding

Funding support for this article was provided by the Wellcome Trust (106593/Z/14/Z).

## Footnotes

1 ACTS Logbook, Irish Family Planning Association Archive (hereafter IFPA Archive). This collection has been deposited at the Royal College of Physicians of Ireland Heritage Centre and will shortly be catalogued.

2 The IFPA by this stage had been in existence for fifteen years, having started out in 1969 as the Fertility Guidance Company and becoming the Irish Family Planning Association in 1972. For more on the history of the IFPA see Laura Kelly, *Contraception and Modern Ireland: A Social History, c.1922–92*, Cambridge, 2023, pp. 184–221.

3 Emilie Cloatre and Mairead Enright, ‘Transformative Illegality: How Condoms “Became Legal” in Ireland, 1991–1993’, *Feminist Legal Studies* 26, 2018, pp. 261–84, p. 268. For an overview of this campaign see Kelly, *Contraception and Modern Ireland*, pp. 304–8.

4 Kelly, *Contraception and Modern Ireland*, pp. 322–3.

5 Ailbhe Smyth, ‘The Women’s Movement in the Republic of Ireland, 1970–1990’, in *Irish Women’s Studies Reader*, ed. Ailbhe Smyth, Dublin, 1993, pp. 245–69, 250.

6 Diarmaid Ferriter, *Ambiguous Republic: Ireland in the 1970s*, London, 2012, p. 679.

7 Interview with John Callaghan, 6 August 2017.

8 Hava Rachel Gordon, ‘Gendered Paths to Teenage Political Participation: Parental Power, Civic Mobility, and Youth Activism’, *Gender and Society* 22: 1, February 2008, pp. 31–55, p. 31.

9 See Sinead McEneaney, ‘Home Sweet Home? Housing Activism and Political Commemoration in Sixties Ireland’, *History Workshop Journal* 87, Spring 2019, pp. 5–26; Laura Kelly, ‘Irishwomen United, the Contraception Action Programme and the Feminist Campaign for Free, Safe and Legal Contraception in Ireland, c. 1975–81’, *Irish Historical Studies* 43: 164, 2019, pp. 269–97; Emilie Cloatre and Mairead Enright, ‘“On the Perimeter of the Lawful”: Enduring Illegality in the Irish Family Planning Movement, 1972–1985’, *Journal of the Law Society* 44: 4, 2017, pp. 471–500, and by the same authors, ‘Transformative Illegality: How Condoms “Became Legal” in Ireland, 1991–1993’, *Feminist Legal Studies* 26, 2018, pp. 261–84.

10 Linda Connolly, *The Irish Women’s Movement: From Revolution to Devolution*, Basingstoke, 2002, and Mary Muldowney, ‘Breaking the Silence: Pro-choice Activism in Ireland since 1983’, in *Sexual Politics in Ireland*, ed. Jennifer Redmond, Sonja Tiernan, Sandra McAvoy and Sonja Tiernan, Dublin, 2015, pp. 127–53. On gay activism, see Patrick McDonagh, *Gay and Lesbian Activism in the Republic of Ireland, 1973–93*, London, 2021; Maurice J. Casey, ‘Radical Politics and Gay Activism in the Republic of Ireland, 1974–1990’, *Irish Studies Review* 26: 2, 2018, pp. 217–36; Ann Nolan, ‘The Gay Community Response to the Emergence of AIDS in Ireland: Activism, Covert Policy, and the Significance of an “Invisible Minority”’, *Journal of Policy History* 30: 1, 2018, pp. 105–27. More broadly, as pointed out recently by Carole Holohan, within the Irish context there has been limited engagement with the history of youth, despite its usefulness ‘as a lens for social historians’; Carole Holohan, *Reframing Irish Youth in the Sixties*, Liverpool, 2018, p. 4.

11 See Caroline Rusterholz’s articles on this subject: ‘Youth Sexuality, Responsibility, and the Opening of the Brook Advisory Centres in London and Birmingham in the 1960s’, *Journal of British Studies* 61: 2, 2022, pp. 315–42; ‘“If we can show that we are helping adolescents to understand themselves, their feelings and their needs, then we are doing [a] valuable job”: Counselling Young People on Sexual Health in the Brook Advisory Centre (1965–1985)’, *Medical Humanities*, 49: 2, 2023, pp. 154–162, and ‘Teenagers, Sex and the Brook Advisory Centres, 1964–85’, in *Children’s Experiences of Welfare in Modern Britain*, ed. Siân Pooley and Jonathan Taylor, London, 2021, pp. 247–71. See also Hannah Charnock, ‘Teenage Girls, Female Friendship and the Making of the Sexual Revolution in England, 1950–1980’, *The Historical Journal* 63: 4, 2020, pp. 1032–53.

12 On emotions and activism, see for example James M. Jasper, *The Emotions of Protest*, Chicago, 2018, and Deborah Gould, *Moving Politics: Emotion and ACT-UP’s Fight against AIDS*, Chicago, 2009.

13 Arlie Russell Hochschild, *The Managed Heart: Commercialization of Human Feeling*, Berkeley, CA, 2012 [1983], p. 20.

14 See Karissa Robyn Patton, ‘“Two more calls, one in tears…”: Emotion, Labour, and Ethics of Care at the Calgary Birth Control Association, 1970–1979’, in *Everyday Health, Embodiment, and Selfhood*, ed. Tracey Loughran, Hannah J. Froom, Kate Mahoney, and Daisy Payling, Manchester, 2024. With thanks to Karissa Patton for sharing her work with me before publication.

15 Gavin Brown and Helen Yaffe, *Youth Activism and Solidarity: The Non-Stop Picket Against Apartheid*, London, 2018, p. 13.

16 Knud Andresen and Bart van der Steen, ‘Introduction: the Last Insurrection? Youth, Revolts and Social Movements in the 1980s’, in *A European Youth Revolt: European Perspectives on Youth Protest and Social Movements in the 1980s*, ed. Knud Andresen and Bart van der Steen, Basingstoke, 2016, p. 2.

17 Andresen and van der Steen, p. 2.

18 Daniel Laqua and Nikolaos Papadogiannis, ‘Youth and Internationalism in the Twentieth Century: An Introduction’, *Social History* 48: 1, 2023, pp. 1–16.

19 Report from IPPF Europe Region Regional Adolescent Services Project Workshop, IFPA Archive.

20 Interview with Siobhán Nowlan, 11 November 2017.

21 Interview with Jon O’Brien, 22 February 2017 (phone).

22 Among the campaigns around contraception in this period were those organized by the Contraception Action Programme, including a caravan which distributed contraceptives and information in disadvantaged communities in Ireland, as well as the earlier work of the Irish Women’s Liberation Movement and their infamous ‘Contraceptive Train’ stunt. See Kelly, *Contraception and Modern Ireland*, pp. 222–60.

23 Cait McKinney, *Information Activism: A Queer History of Lesbian Media Technologies*, Durham, North Carolina, 2020, p. 78.

24 Hannah Zeavin, *The Distance Cure: A History of Teletherapy*, Cambridge, MA, 2021, pp. 93–128.

25 McKinney, *Information Activism*, p. 84.

26 Zeavin, *The Distance Cure*, p. 99.

27 Terri Chattiar, ‘Counselling for Connection: Making Queer Relationships during Britain’s Sexual Revolution’, *Medical Humanities*, 49: 2, 2023, pp. 182–92.

28 McKinney, *Information Activism*, p. 84. On telephone support hotlines see also George Severs, ‘Male Rape: Survivors, Support and the Law in Late Twentieth-Century England and Wales’, *History Workshop Journal* 97, pp. 196–220.

29 Rusterholz, ‘Teenagers, Sex and the Brook Advisory Centres’, p. 252.

30 Valuable exceptions include Kate Fisher, *Birth Control, Sex and Marriage in Britain, 1918–1960*, Oxford, 2006; Kate Fisher and Simon Szreter, *Sex Before the Sexual Revolution: Intimate Life in England, 1918–1963*, Cambridge, 2010; and Katherine Jones, ‘“Men Too”: Masculinities and Contraceptive Politics in Late Twentieth Century Britain’, *Contemporary British History* 34: 1, 2020, pp. 44–70.

31 Participants were contacted through snowballing after an initial interview with Christine Donaghy, who had been information and education officer at the IFPA. Interviews generally took place at the participant’s home or a mutually convenient location such as a café. Interviewees were sent a copy of their interview transcript to make any corrections following the interview. With participants’ permission, interview recordings and/or transcripts from the bigger project will shortly be deposited at the Scottish Oral History Centre collection at the University of Strathclyde.

32 For further discussion of the Family Planning Act see Aidan Beatty, ‘Irish Modernity and the Politics of Contraception, 1979–1993’, *New Hibernia Review* 17: 3, 2013, pp. 100–18, and Brian Girvin, ‘An Irish Solution to an Irish Problem: Catholicism, Contraception and Change, 1922–1979’, *Contemporary European History* 27, March 2018, pp. 1–22.

33 Kelly, *Contraception and Modern Ireland*, p. 300.

34 Chrystel Hug, *The Politics of Sexual Morality in Ireland*, Basingstoke, 1999, p. 122.

35 Cloatre and Enright, ‘Transformative Illegality’, p. 269.

36 Jon O’Brien, *Young People & Family Planning: The Learning Experience of the Irish Family Planning Association, 1984–1990* (1990 project report), p. 1.

37 Kelly, *Contraception and Modern Ireland*, pp. 293–304.

38 Mandatory sex education was not introduced into Irish schools until 1997, with the introduction of the Relationships and Sexuality Programme. See Ann Nolan, ‘The Transformation of School-Based Sex Education Policy in the Context of AIDS in Ireland’, *Irish Educational Studies*, 37: 3, 2018, pp. 295–309.

39 ‘Life Line’, *In Dublin*, March 20, 1986, pp. 7–8, IFPA Archive.

40 For an outline of the history of the Pro-Life Amendment Campaign and its 1983 referendum campaign, see Tom Hesketh, *The Second Partitioning of Ireland?: The Abortion Referendum of 1983*, Dublin, 1990.

41 Hug, *Politics of Sexual Morality*, p. 121.

42 See Nell McCafferty, *A Woman to Blame: the Kerry Babies Case*, Dublin, 1985.

43 Moira J. Maguire, ‘The Changing Face of Catholic Ireland: Conservatism and Liberalism in the Ann Lovett and Kerry Babies Scandals’, *Feminist Studies*, 27: 2, 2001, pp. 335–58, p. 338.

44 Patrick McDonagh, *Gay and Lesbian Activism in the Republic of Ireland, 1973–93*, London, 2021, p. 22.

45 McDonagh, *Gay and Lesbian Activism*, pp. 36, 62.

46 Mary Gordon, ‘Fighting for Control – the Ongoing Struggle for Reproductive Rights’, *The Irish Feminist Review*, Volume 1, 1984, pp. 7–29, 10.

47 Interview with Miriam Watchorn, 7 November 2018.

48 Interview with John Callaghan, 6 August 2017.

49 James M. Jasper, *The Emotions of Protest*, Chicago 2018, p. 46.

50 Interview with Jon O’Brien.

51 Interview with Jon O’Brien.

52 Interview with John Callaghan.

53 Interview with Miriam Watchorn.

54 Interview with Siobhán Nowlan.

55 Interview with Jon O’Brien.

56 Interview with John Callaghan.

57 O’Brien, *Young People & Family Planning*, p. 6.

58 O’Brien, *Young People & Family Planning*, p. 16.

59 Damian Corless, ‘Sexual healing’, *Hot Press*, 9: 3, 1 March 1985, p. 21.

60 O’Brien, *Young People & Family Planning*, pp. 16–17, p. 8.

61 O’Brien, *Young People & Family Planning*, p. 7.

62 Memo: Youth Group Training 1988 (9963), IFPA Archive.

63 Jon O’Brien, *Irish Family Planning Association Pilot Project Report, Nov ‘86/April ’87: Young People’s Family Planning Centre*, 1987, p. 10.

64 Interview with Finn van Gelderen, 20 October 2017.

65 O’Brien, *Young People & Family Planning*, pp. 18–19, and author’s correspondence with Jon O’Brien.

66 O’Brien, *Young People & Family Planning*, p. 9.

67 Interview with Siobhán Nowlan.

68 Telephone answering techniques, training session 1, IFPA Archive.

69 O’Brien, *Young People & Family Planning*, p. 20.

70 Interview with John Callaghan.

71 See Rusterholz, ‘Youth Sexuality, Responsibility, and the Opening of the Brook Advisory Centres’, pp. 330–9.

72 Hug, *Politics of Sexual Morality*, p. 116. TD (Teachta Dála) is the Irish equivalent of MP, member of parliament.

73 ACTS Logbook, 8 February 1985, IFPA Archive.

74 ‘Angela hits at hot line on sex advice’, *Sunday Independent*, December 23, 1984. (Folder of newspaper clippings, IFPA Archive).

75
*Response*, 4: 3, Autumn 1985.

76
*Response*, 3: 4, Winter 1984.

77
*Response*, 4: 1, Spring 1985.

78 ‘Clinic to open despite threat’, *Irish Times*, October 31, 1986, p. 8.

79 Interview with Miriam Watchorn.

80 Interview with John Callaghan.

81 Interview with Jon O’Brien.

82 O’Brien, *Young People & Family Planning*, p. 5.

83 Jon O’Brien, ‘The story of ACTS and SECSI, Irish FPA Youth groups’, *Make it happen, make it now: young people’s involvement in family planning associations*, European Youth Strategy working group report, 1996, https://wellcomecollection.org/works/hxscfngd.

84 Letter from executive committee member to Jon O’Brien, 12 June 1986, IFPA Archive.

85 Jon O’Brien, *Irish Family Planning Association Pilot Project Report, Nov ‘86/April ’87: Young People’s Family Planning Centre*, 1987, p. 10.

86 O’Brien, *IFPA Pilot Project Report*, p. 10.

87 O’Brien, *Young People & Family Planning.*

88 ACTS Logbook, 2 April 1988, IFPA Archive.

89 ACTS Logbook, 10 June 1989, IFPA Archive.

90 Kelly, *Contraception and Modern Ireland*, p. 18.

91 IFPA Youth Group AGM Minutes, 23 December, 1986, IFPA Archive.

92 ‘Family group indicts sexual myths’, *Irish Independent*, 11 March, 1986, p. 3.

93 ‘Life Line’, *In Dublin*, March 20, 1986, pp. 7–8, IFPA Archive.

94 Interview with Gerry Curran, 20 October 2017.

95 O’Brien, *Young People & Family Planning.*

96 ACTS Logbook, Midweek calls, February 1986, IFPA Archive.

97 Interview with Siobhán Nowlan.

98 ACTS Logbook, 21 January 1986, IFPA Archive.

99 ACTS Logbook, 2 March 1985, IFPA Archive.

100 O’Brien, *Young People and Family Planning*.

101 The population of Dublin in 1981 was 915,115 people, while the population of Ireland was 3,453 million. Kevin Hourihan, ‘Population Redistribution in Irish Cities: Dublin, Cork and Limerick, 1971–1981’, *Irish Geography* 16: 1, 1983, pp. 113–20. O’Brien, *Young People & Family Planning*, p. 8.

102 ‘Life Line’, *In Dublin*, March 20 1986, pp. 7–8, IFPA Archive.

103 ACTS Logbook, 1 February 1985, IFPA Archive.

104 ACTS Logbook, 12 January 1985, IFPA Archive.

105 ‘Life Line’, *In Dublin*, March 20, 1986, pp. 7–8, IFPA Archive.

106 Telephone answering techniques, training session 1, IFPA Archive.

107 ACTS Logbook. 26 March 1988, IFPA Archive.

108 ACTS Logbook, 2 October 1985 and 1 December 1984, IFPA Archive.

109 Interview with Finn van Gelderen.

110 ACTS Logbook, 21 February 1987, IFPA Archive.

111 ‘Life Line’, *In Dublin*, March 20, 1986, pp. 7–8, IFPA Archive.

112 ACTS Logbook, 2 July 1988, IFPA Archive.

113 ACTS Logbook, 10 November 1984, IFPA Archive.

114 Interview with John Callaghan.

115 On women’s experiences of illegal abortion in Ireland in the early twentieth century, see Cara Delay, ‘Pills, Potions and Purgatives: Women and Abortion Methods in Ireland, 1900–1950’, *Women’s History Review* 28: 3, 2019, pp. 479–99; ‘Kitchens and Kettles: Domestic Spaces, Ordinary Things, and Female Networks in Irish Abortion History, 1922–1949’, *Journal of Women’s History* 30: 4, 2018, pp. 11–34, and ‘“With Foolish Optimism We Thought it was Just a Miss”: Marriage, Sexuality, and Unwanted Pregnancies in Twentieth-Century Ireland’, *Review of Irish Studies in Europe* 5: 1, 2022, pp. 28–42. For accounts of women’s experiences of travelling to England for abortions, see Erin Darcy, *In Her Shoes: Women of the Eighth: A Memoir and Anthology*, Dublin: New Island Books, 2020, and Medb Ruane, *The Irish Journey: Women’s Stories of Abortion*, Dublin: Irish Family Planning Association, 2000.

116 ACTS Logbook, 14 August 1985, IFPA Archive.

117 ACTS Logbook, 6 February 1988, IFPA Archive.

118 ACTS Logbook, 1 February 1985, IFPA Archive.

119 ACTS Logbook, 31 May 1986, IFPA Archive.

120 Interview with Miriam Watchorn.

121 ACTS Logbook, 8 November 1986, IFPA Archive.

122 ACTS Logbook, 15 November 1986, IFPA Archive.

123 ACTS Logbook, 3 March 1990, IFPA Archive.

124 Interview with Finn van Gelderen.

125 ACTS Logbook, 27 April 1985, IFPA Archive.

126 ACTS Logbook, 26 January 1985, IFPA Archive.

127 ACTS Logbook, 4 May 1985, IFPA Archive.

128 ACTS Logbook, 10 August 1985, IFPA Archive.

129 ACTS Logbook, 19 September 1987, IFPA Archive.

130 ACTS Logbook, 20 March 1985, IFPA Archive.

131 ACTS Logbook, 2 August 1986, IFPA Archive.

132 McKinney, *Information Activism*, p. 82.

133 Interview with Finn van Gelderen.

134 Interview with Miriam Watchorn.

135 ACTS Logbook, 18 February 1985, IFPA Archive.

136 ACTS Logbook. 23 February 1985, IFPA Archive.

137 ‘Sexual healing’, *Hot Press*, 9: 3, 1 March 1985, p. 22.

138 Ursula Barry, ‘Abortion in the Republic of Ireland’, *Feminist Review* 29, Summer 1988, pp. 57–63, p. 60.

139 Interview with Siobhán Nowlan.

140 ACTS Logbook, 6 June 1987, IFPA Archive.

141 ACTS Logbook, 11 February 1989, IFPA Archive.

142 ACTS Logbook. 4 October 1986, IFPA Archive.

143 ACTS Logbook, 3 January 1987, IFPA Archive.

144 ACTS Logbook, 30 April 1988, IFPA Archive.

145 ACTS Logbook, 10 February 1990, IFPA Archive.

146 Interview with John Callaghan.

147 ACTS Logbook, 24 May 1986, IFPA Archive.

148 ACTS Logbook, 24 May 1986, IFPA Archive.

149 ACTS Logbook, 11 October 1986, IFPA Archive.

150 Interview with John Callaghan.

151 Interview with Gerry Curran.

152 McKinney, *Information Activism*, p. 68.

153 Gould, *Moving Politics*, p. 209.

154 Lorraine Grimes, Joanna Mishtal, Karli Reeves, Dyuti Chakravarty, Bianca Stifani, Wendy Chavkin, Deirdre Duffy, Trish Horgan, Mary Favier, Mark Murphy, Antonella F. Lavelanet, ‘“Still Travelling”: Access to Abortion Post-12 Weeks Gestation in Ireland’, *Women’s Studies International Forum*, Volume 98, May–June 2023, pp. 1–8.

155 Joanna Mishtal, Karli Reeves, Dyuti Chakravarty, Lorraine Grimes, Bianca Stifani, Wendy Chavkin and others, ‘Abortion Policy Implementation in Ireland: Lessons from the Community Model of Care’, *PLoS* ONE, 17: 5, 2022, pp. 1–23.

## Author Biography

**Laura Kelly** is professor of modern Irish history at the University of Strathclyde, Glasgow. Her most recent book, *Contraception and Modern Ireland: A Social History, c. 1922–92*, was published by Cambridge University Press in 2023. Her research focuses on the social history of medicine in Ireland, gender history, and the history of activism.

